# COVID-19 outbreaks caused by different SARS-CoV-2 variants: a descriptive, comparative study from China

**DOI:** 10.3389/fpubh.2024.1416900

**Published:** 2024-12-12

**Authors:** Cao Chen, Yenan Feng, Zeyuan Yin, Mingfan Pang, Qi Shi, Xuejun Ma, Xiao-Ping Dong

**Affiliations:** ^1^National Key Laboratory of Intelligent Tracking and Forecasting for Infectious Diseases, NHC Key Laboratory of Medical Virology and Viral Diseases, Collaborative Innovation Center for Diagnosis and Treatment of Infectious Diseases (Zhejiang University), National Institute for Viral Disease Control and Prevention, Chinese Center for Disease Control and Prevention, Beijing, China; ^2^Center for Biosafety Mega-Science, Chinese Academy of Sciences, Wuhan, China; ^3^Center for Global Public Health, Chinese Center for Disease Control and Prevention, Beijing, China; ^4^China Academy of Chinese Medical Sciences, Beijing, China; ^5^Shanghai Institute of Infectious Disease and Biosafety, Shanghai, China

**Keywords:** COVID-19, SARS-CoV-2, outbreak, variants, omicron

## Abstract

**Objectives:**

To understand the epidemic characteristics of various SARS-CoV-2 variants, we mainly focus on analyzing general epidemic profiles, viral mutation, and evolution of COVID-19 outbreaks caused by different SARS-CoV-2 variants of concern (VOCs) in China as of August 2022.

**Methods:**

We systematically sorted out the general epidemic profiles of outbreaks caused by various SARS-CoV-2 VOCs in China, compared the differences of outbreaks caused by Delta and Omicron VOCs, and analyzed the mutational changes of subvariants between the same outbreak and different outbreaks.

**Findings:**

By 15 August 2022, a total of 2, 33, and 124 COVID-19 outbreaks caused by Alpha, Delta, and Omicron VOCs, respectively, were reported in different regions of China. In terms of the number of outbreaks, the extent of affected areas, and the total number of confirmed cases, Omicron VOCs were more widespread than the other variants. The most frequently circulating PANGO lineages in China were B.1.617.2 and AY.122 in Delta VOCs, and BA.2.2.1, BA.2, BA.2.2, and BA.5 for Omicron VOCs. Additional mutations in the genome of the SARS-CoV-2 strain were frequently observed in outbreaks with longer duration and higher numbers of infections.

**Conclusion:**

Through the comprehensive analysis of the COVID-19 outbreaks, the influences, and the evolution of the SARS-CoV-2 variants in China, we found differences between outbreaks caused by Delta and Omicron VOCs. The genome of SARS-CoV-2 continued to evolve within the same outbreak and across outbreaks occurring in different locations or at different times. These findings suggest that rapidly containing an Omicron virus outbreak can not only reduce the spread of the virus but also delay the virus’s mutation frequency.

## Introduction

According to the World Health Organization (WHO), the coronavirus disease 2019 (COVID-19) pandemic has affected more than 220 countries and regions, resulting in over 776 million confirmed cases and 7.06 million deaths. The pandemic has caused an unprecedented impact on global health and global economic development (1). The ongoing evolution of severe acute respiratory syndrome coronavirus 2 (SARS-CoV-2), the causative agent of COVID-19 (2), continually generates new variants with enhanced transmissibility, increased clinical severity, and/or the ability to evade immunity. These variants have led to multiple epidemic peaks worldwide (3–6). Since the emergence of COVID-19 in 2020, the WHO has designated several variants as the variant of concern (VOC) and variant of interest (VOI) (4).

The Omicron variant was identified as VOC at the end of 2021 (7). Since then, Omicron VOCs have revealed faster evolution and mutation speed, leading to remarkably faster and wider transmissions globally than the ancestral VOCs (Alpha, Beta, Gamma, and Delta) (7, 8). Different Omicron subvariants (Phylogenetic Assignment of Named Global Outbreak Lineages BA.1, BA.2, and BA.5) have progressively replaced the original strains, becoming the dominant variants in the ongoing pandemic (9).

Omicron VOC-associated outbreak in China was identified at the beginning of 2022 and became the only endemic strain afterward, with similar transmission characteristics as the rest of the world (10). In order to understand the epidemic characteristics, especially in the general epidemic profiles, viral mutation, and evolution of various SARS-CoV-2 variants better in China, we summarized the general patterns of domestic outbreaks of various SARS-CoV-2 VOCs in China, compared the differences in the outbreaks caused by Delta and Omicron VOCs, and analyzed the mutational changes of subvariants in the same outbreak and between different outbreaks.

## Materials and methods

### Data collection

COVID-19 outbreak in this study is the event containing 5 and > 5 confirmed cases that shared the epidemiological and viral genomic relation in a prefectural-level administrative region in China. Considering the comprehensive nature of data information, data were collected from 17 January 2021, when the first case of Alpha VOC was identified to 15 August 2022. The data on the outbreaks, including virus strains, durations, numbers of involved cases, etc., were collected from the China CDC. The data of the whole genomes of SARS-CoV-2 viruses involved in each outbreak were obtained from the National Institute for Viral Disease Control and Prevention (IVDC), China CDC. Relevant COVID-19 epidemiological data were obtained from the Global Epidemic Analysis and Risk Assessment Platform, China CDC (11).

### Bayesian phylogenetic analysis

BEAST v.1.10.4 was employed for evolutionary rates analysis. We utilized a general time-reversible substitution model with gamma-distributed rate variation among sites, an Uncorrelated Relaxed Clock with a lognormal relaxed distribution, and an exponential growth coalescent tree prior. The Markov Chain Monte Carlo (MCMC) chains comprising 100 million states were merged. Sampling of parameters and trees occurred every 10,000 steps, discarding the initial 10% as burn-in. To ensure an effective sample size >200 for all estimated parameters, convergence and mixing of MCMC chains were assessed using Tracer v.1.7.2. Maximum clade credibility (MCC) trees were summarized using TreeAnnotator v.1.10.4 and visualized with FigTree v.1.4.4.

### Maximum-likelihood phylogenetic tree

The representative sequences were then aligned against the reference (NC_045512.2) using the Nextclade (v 3.5.0) tool to create a consensus alignment file. For phylogenetic analyses, the maximum-likelihood phylogeny of the sequences was determined using IQ-TREE (v2.1.4) with the best-fitting substitution model chosen by the Bayesian Information Criterion (BIC). To visualize the phylogenetic trees, we used FigTree software in the present study (v1.4.4).

### Statistical analysis

Analysis of the numbers of subvariants with infected cases and durations in a single Omicron outbreak in China was carried out using GraphPad Prism 9.0. The *p*-values for differences among the three groups were determined using the Kruskal–Wallis test.

## Results

### The general patterns of the domestic outbreaks caused by various SARS-CoV-2 VOCs in China

**Figure 1:**
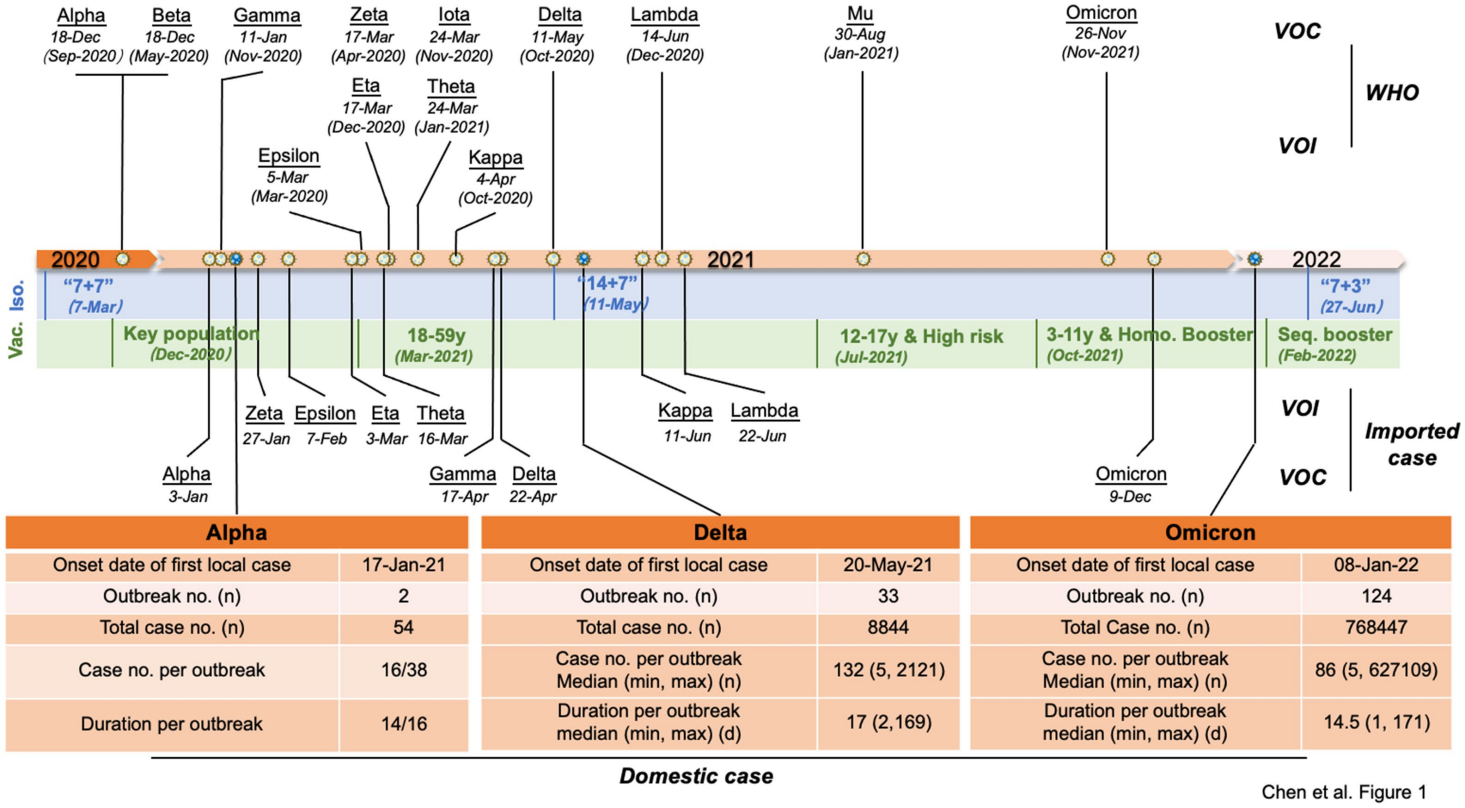
Timeline of the VOCs and VOIs of SARS-CoV-2 and COVID-19 control policies and vaccination strategies in China. The designated dates of VOCs and VOIs are marked on the top of the timeline with the earliest documented date indicated in parentheses, and the times of the imported VOCs and VOIs in China are indicated below. The primary COVID-19 control policies (blue areas) and vaccination strategies (green areas) in China at different time periods are listed below the timeline. Key populations encompass individuals involved in import cold chain management, port quarantine operations, ship pilotage services, aviation aircrew personnel, fresh market workers, public transport employees, medical disease control professionals, and other industries characterized by a high risk of infection. Additionally, this includes individuals traveling for work or study purposes to countries or regions classified as medium or high risk. High-risk groups primarily consisted of individuals aged 60 years and above as well as those with pre-existing medical conditions. Booster represents homologous booster vaccination, which refers to receiving a booster dose of the same vaccine type as the initial doses. Seq. booster represents sequential (or heterologous) booster vaccination, which involves receiving a booster dose of a different vaccine type from the initial doses. Major epidemiological data of Alpha-, Delta-and Omicron-associated outbreaks in China are summarized in the table below the timeline. Abbreviation: Iso = Isolation; Vac = vaccination; “7 + 7” = 7-day isolation in designated hotel +7 home health monitoring; “14 + 7” = 14-day isolation in designated hotel +7 home health monitoring; “7 + 3” =7-day isolation in designated hotel +3 home health monitoring.

Since the WHO designated SARS-CoV-2 VOCs and VOIs at the end of 2020, the cases infected with different VOCs or VOIs were continually identified in China. Except for 3 outbreaks caused by non-VOCs in early 2021, all local outbreaks (5 and > 5 confirmed cases) were associated with VOCs Alpha, Delta, and Omicron. Beta VOC caused only one local transmission involving two cases. As shown in Figure 1, the first VOC Alpha-, Delta-and Omicron-induced outbreaks in China occurred on 17 January 2021, 20 May 2021, and 08 January 2022, respectively. As of 15 August 2022, 2, 33, and 124 local outbreaks caused by Alpha, Delta, and Omicron variants were reported in China, causing 54, 8,848, and 768,447 cases, respectively. The involved case numbers of those outbreaks varied largely from 5 to 2,121 cases (median: 132) in Delta outbreaks and 5 to 627,109 cases (median: 86) in Omicron outbreaks. The duration of outbreaks (the reported date from the first to last case) also varied dramatically. The duration of the two Alpha outbreaks was 14 and 16 days, while the duration of Delta outbreaks ranged from 2 to 169 days (median: 17), and Omicron outbreaks lasted from 1 to 171 days (median: 14.5), respectively.

**Figure 2:**
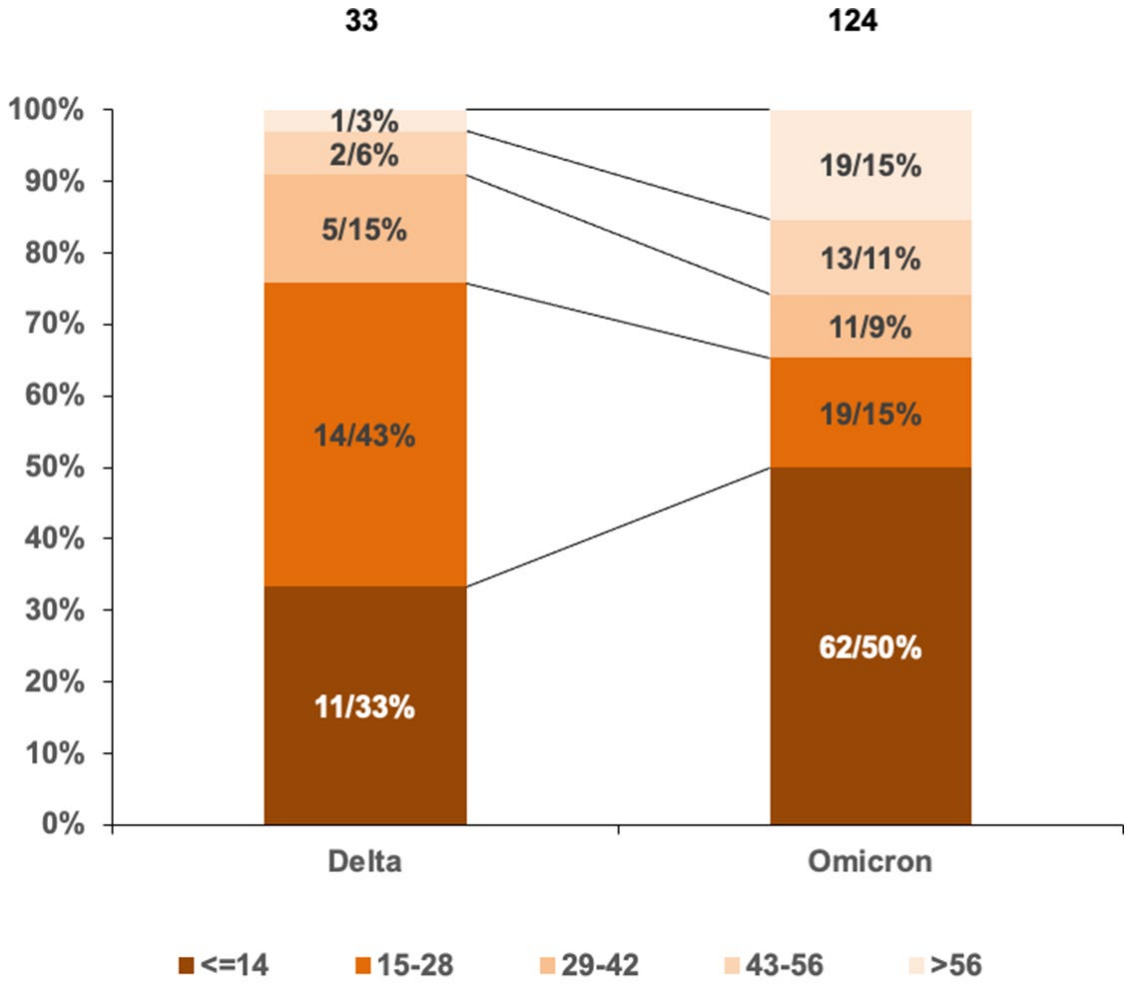
Proportions in the lengths of outbreak durations induced by Delta and Omicron VOCs in China. Grouping the lengths of outbreak durations is based on the maximal incubation period of COVID-19 (14 days). The total numbers of Delta and Omicron outbreaks are indicated above. The numbers and percentages of Delta and Omicron outbreaks in various groups are marked inside columns. Various intervals are distinguished by different colors.

**Figure 3:**
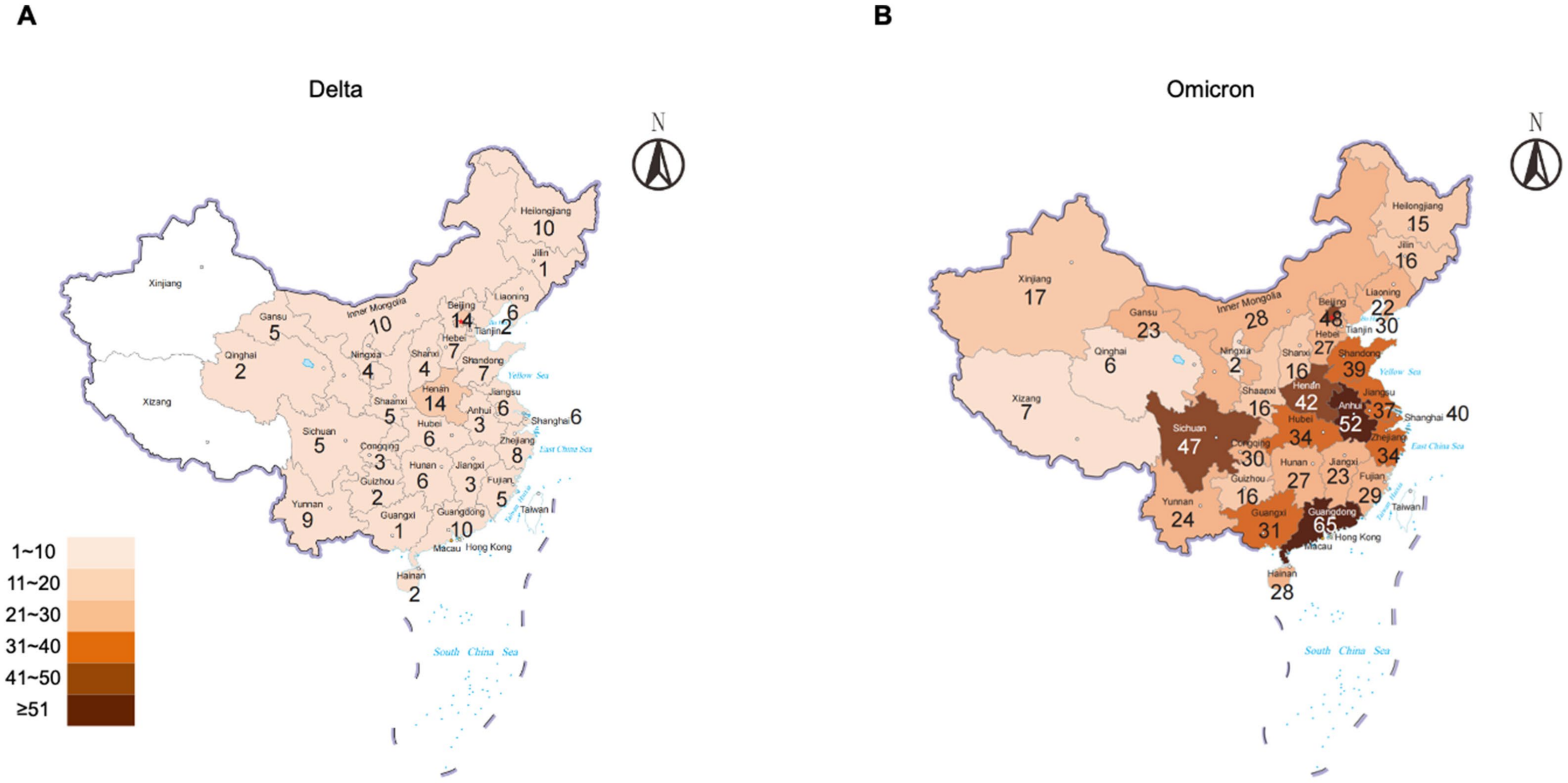
Numbers of Delta and Omicron outbreaks that affected the regions of the prefectural level in the context of the provinces of China. **(A)** Delta. **(B)** Omicron. The total numbers of the outbreaks are indicated inside each province.

The durations of Delta and Omicron outbreaks in China showed different patterns; 91% (30/33) of Delta outbreaks ended with relatively short duration (shorter than three maximal incubation periods, 42 days) in which 33% ended within 14 days, 43% within 28 days, and 15% within 42 days, while 74% (92/124) of Omicron outbreaks ended within 42 days (50% within 14 days, 15% within 28 days, and 9% within 42 days) (Figure 2). The impact of Omicron was more pronounced than that of Delta for outbreaks lasting longer than three maximal incubation periods; 26% (32/124) of Omicron outbreaks (11% within 56 days and 15% longer than 56 days) and 9% (3/33) of Delta ones (6% within 56 days and 3% longer than 56 days) lasted with long duration (Figure 2). The spreading areas in the context of administrative regions (provincial level or prefectural level) of the outbreaks also differed. One Alpha outbreak affected one individual city in two provinces, and the other involved only one city in one province; 33 Delta outbreaks overflowed and affected 166 times at the prefecture-level administrative regions of 29 provinces in China (Figure 3A), whereas 124 Omicron outbreaks affected 871 times at the prefectural-level regions of 31 provinces (Figure 3B).

### Different profiles of the outbreaks caused by Delta and omicron subvariants

**Figure 4:**
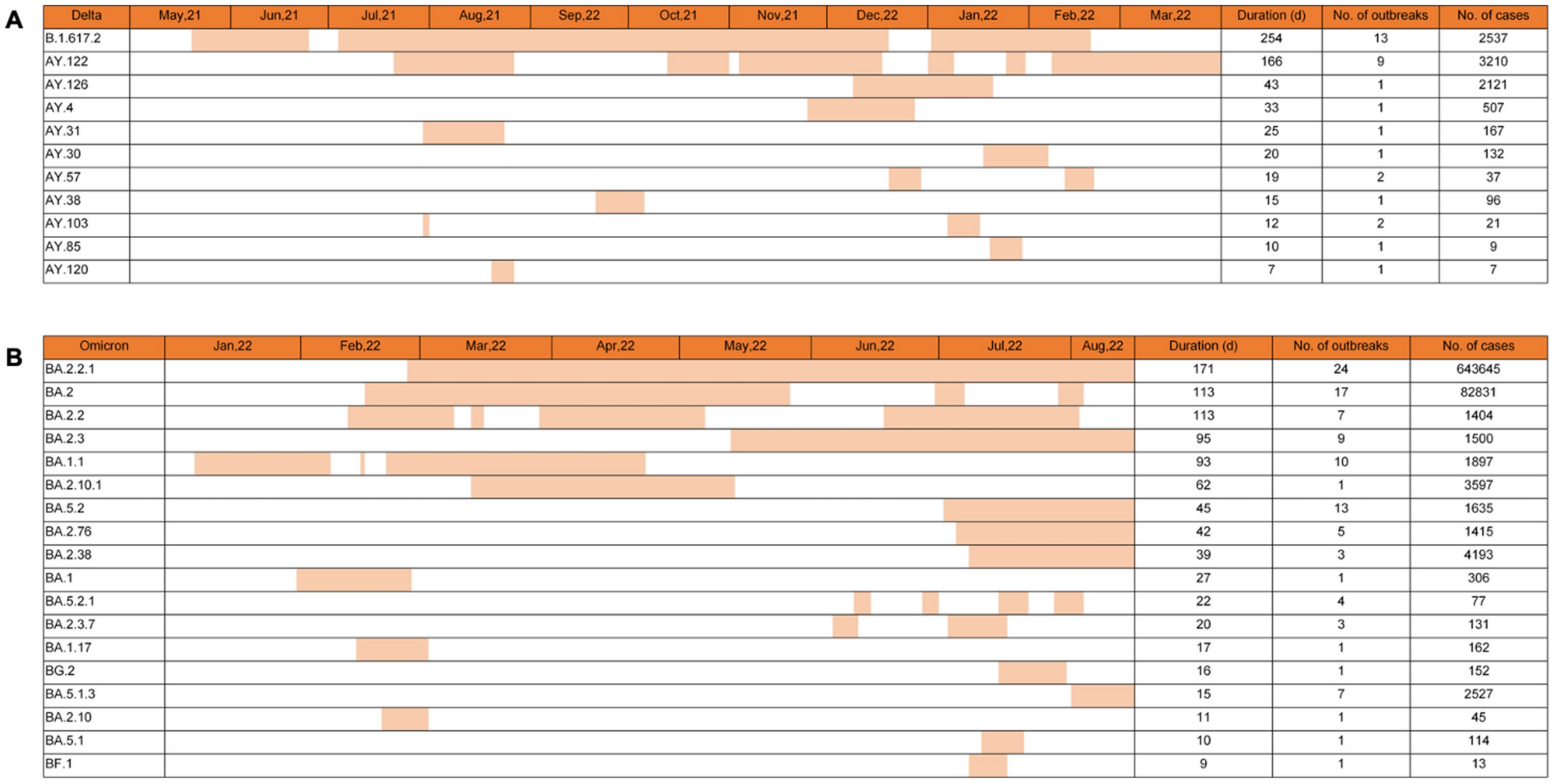
Epidemic times of various PANGO lineage subvariants of Delta and Omicron VOCs in China. The epidemic times of 10 subvariants of Delta VOCs **(A)** and 18 subvariants of Omicron VOCs **(B)** are illustrated. The general durations, the numbers of the associated outbreaks, and the numbers of the confirmed cases of various subvariants are summarized on the right. Corresponding data are indicated at the top of each graph.

The spreading features of VOCs Delta and Omicron-induced outbreaks were further analyzed based on the subvariants of Phylogenetic Assignment of Named Global Outbreak (PANGO) Lineages (11). The total epidemic duration of VOC Delta in China was 315 days, from the first case (B.1.617.2) on 20 May 2021 to the last one (AY.122) on 31 March 2022 (Figure 4A). Delta subvariants B.1.617.2 and AY.122 revealed the longest epidemic times in the context of China, lasting 254 and 166 days with different times of intermissions, which induced 13 and 9 outbreaks and caused 2,537 and 3,210 cases, respectively. Subvariant AY.126 caused only one outbreak but affected 2,121 cases with a duration of 43 days. The rest subvariants, including AY.4, AY.31, AY.30, AY.57, AY.38, AY.103, AY.85, and AY.120, showed shorter durations (7–33 days), involving one or two outbreaks with the smallest number of seven cases and the largest of 507 cases.

By 15 August 2022, 124 Omicron outbreaks were reported in China, involving at least 18 subvariants. Based on the data of virus genome sequences in each outbreak, 71 out of 124 (57.3%) outbreaks were caused by a single virus strain, 38 (30.6%) were significantly associated with a predominant virus strain that was identified in >60% obtained virus sequences, and 15 (12.1%) were mixed with 2 or > 2 different virus strains in which the difference in the proportion between various was less than 50%. The numbers, durations, and involved cases of the outbreaks caused by a single strain or by a predominant strain were selected and counted. As shown in Figure 4B, the longest three epidemic times were induced by subvariants BA.2.2.1, BA.2, and BA.2.2, with 171, 113, and 113 days, respectively. The largest three case numbers were associated with BA.2.2.1, BA.2, and BA.2.38, with total case numbers of 643,645, 82,831, and 4,193. The most three outbreak numbers were caused by BA.2.2.1, BA.2, and BA.5.2, with outbreak numbers of 24, 17, and 13, respectively. In particular, subvariant BA.2.2.1 was the SARS-CoV-2 strain that induced the most outbreak number (24 outbreaks), infected the largest case number (643,645 cases), and lasted the longest circulating time (117 days) in China. By 15 August 2022, subvariants BA.2.2.1, BA.2.3, BA.5.2, BA.2.76, BA.2.38, and BA.5.1.3 still circulated in China.

**Figure 5:**
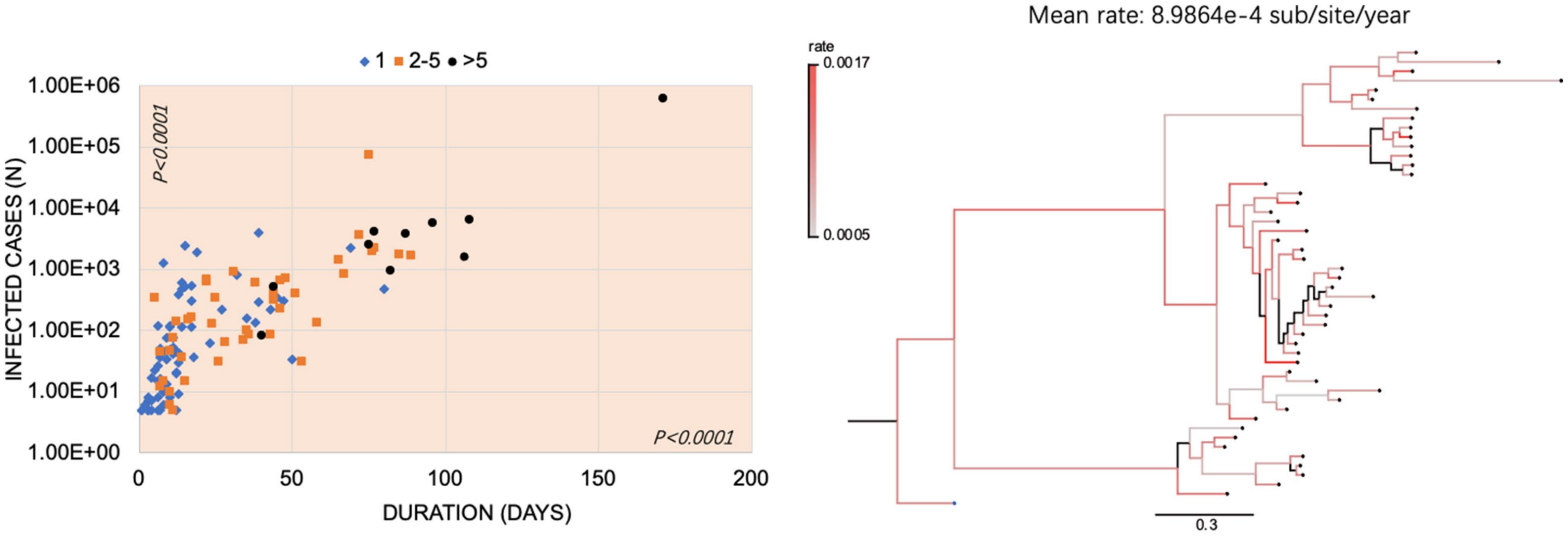
The Omicron outbreaks in China. **(A)** Relationship of the number (s) of subvariants with infected cases and durations in Omicron outbreaks in China. The x-axis represents the duration (day); the y-axis represents the number of infected cases and is expressed in the format with lg. The outbreaks with 1 subvariant, 2–5 subvariants, and > 5 subvariants were marked with blue diamonds, orange squares, and black circles, respectively. *p*-values were indicated in each axis. **(B)** The mean rate of evolution and phylogenetic analysis of the representative sequences was determined using Beast. The color-coded branches indicate the rate of each branch. The scale bar indicates the genetic distance between sequences.

To evaluate the possible correlation among the numbers of subvariants, the confirmed cases, and the duration of the outbreaks, 124 Omicron outbreaks were divided into three groups: 71 outbreaks with one single subvariant, 43 outbreaks with 2 to 5 subvariants, and 10 outbreaks with >5 subvariants, respectively. As shown in Figure 5, the overwhelming majority of the outbreaks with one single subvariant had a short duration (<50 days, 95.8% (68/71)) and fewer case numbers (<1,000 cases, 93.0% (66/71)). In the group of the outbreaks with two to five subvariants, 25.6% (11/43) were in the duration of 51–100 days and 16.3% (7/43) had more than 1,000 cases. In the group of outbreaks with >5 subvariants, eight out of ten (80.0%) lasted for a long time (>50 days) and had more cases (>1,000). Statistical analysis revealed significant differences (*p* < 0.0001) in the numbers of subvariants, infections, and durations among these three groups. Based on Bayesian phylogenetic analysis, the estimated evolutionary rate was 8.9864e-4 sub/site/year (95% highest posterior density interval 5.8089e-4 − 1.2479e-3).

### Increase of additional mutations in the same subvariants of PANGO lineage within the same outbreaks and among the different outbreaks

To calculate the further mutation trends of a special subvariant in one outbreak in one place, the numbers of nucleotide exchanges in the viral whole genome sequences from the early and late stages of the outbreak were counted. As summarized in Supplementary Table S1, in the case of Omicron VOCs, the subvariant BA.2.2.1 outbreak in Shanghai lasted 171 days and infected 627,109 cases. Ten additional mutations were identified in the viral genomes obtained in the late stage compared to that of the early stage. The outbreaks of BA.2, BA.2.3, and BA.1.1 in Liaoning, Guangxi, and Tianjin lasted for 77 days with 4,192 cases, 35 days with 161 cases, and 31 days with 905 cases, whereas four, three, and two new mutations were observed in the viruses at the late stages, respectively. For the Delta VOCs, three, three, two, and one additional mutations were identified in the viruses at late stages of the outbreaks of AY.126, B.1.617.2, AY.122, and AY.31 in Shaanxi, Fujian, Jiangsu, and Henan provinces, which lasted for 43 days with 2,121 cases, 23 days with 471 cases, 37 days with 1,162 cases, and 25 days with 167 cases, respectively. It highlights that the longer lasting time and more numbers of infected cases are associated with more additional mutations of the viruses.

We also noticed that some Omicron outbreaks in China were caused by the same subvariant of PANGO lineage at least at 3-month intervals but without identifiable epidemiological association. Supplementary Table S2 summarizes the onset dates and viral mutation data of some Omicron outbreaks in China. Subvariant BA.2.2.1 was the predominant strain in the Beijing outbreak during April 2022, which contained 74 point mutations compared to the original strain. BA.2.2.1 was also identified as the predominant virus strain in the outbreak in Hubei 3 months later, containing four additional mutations located at the ORF1a region. Similarly, subvariant BA.2.2 was the predominant strain in the Shenzhen outbreak during February 2022, which contained 69 point mutations compared to the original strain. BA.2.2 was also identified as the predominant virus strain in the Shenzhen outbreak 3 months later, containing seven additional mutations located at ORF1a, ORF1b, and ORF3a region. The phylogenetic analysis showed that both BA.2.2 and BA.2.2.1 differentiated into several clades (Supplementary Figure S1). Moreover, the earliest BA.2.2 or BA.2.2.1 sublineage of the earliest local outbreak only appeared in one of the clades, indicating that even within the same BA.2.2 or BA.2.2.1 lineage, the outbreak strains caused by this lineage in China have diverse origins and may stem from multiple independent introductions, rather than evolving solely within China.

These results indicate that the genomes of SARS-CoV-2 continually evolve within the period of the same outbreak and among the outbreaks that occurred at different places or times.

## Discussion

Since the emergence of COVID-19, thousands of PANGO lineage subvariants and dozens of WHO-designated SARS-CoV-2 variants have been proposed based on viral whole genome sequences (12, 13). Some of them became pandemic-dominant strains, caused millions of infections, and persisted for long times, whereas the majority of them appeared to be prevented locally and became less detectable in a relatively short period (6, 8). In this study, we have, for the first time, comprehensively analyzed the features of the COVID-19 outbreaks caused by different SARS-CoV-2 variants in China and figured out the diversity of the different outbreaks in the context of WHO-designated VOCs and PANGO sub-lineages.

Our data here indicate that Alpha, Delta, and Omicron VOCs have induced local COVID-19 outbreaks in China, although there have been sporadic cases or small clusters (less than five cases) induced by VOCs/VOIs or other non-VOCs/VOIs that were associated with the imported cases with definite epidemiological clues. Coinciding with the global prevalence periods of Alpha, Delta, and Omicron VOCs, corresponding outbreaks in China occurred at the same periods. However, the Alpha-, Delta-, and Omicron-associated outbreaks in China revealed completely different profiles, regardless of the outbreak numbers, the affecting or overflowing areas, or confirmed cases, in which Omicron VOCs are significant. Considering the similar prevalence phases of Alpha (approximately 6 months), Delta (approximately 7 months), and Omicron (approximately 8 months until August 2022), and the almost unchanged COVID-19 control policy in China, one may assume that the marked diversities of those three VOCs in inducing local transmission and outbreaks are largely due to the difference in virus biology, apparently reflecting upon the viral genomes.

Contrary to the original strain and other earlier VOCs, Delta and Omicron VOCs demonstrate higher transmissibility (14). Compared to the Delta VOC, Omicron VOC has caused significantly more outbreaks and affected more cases in different regions of China within 8 months (from January to August 2022), including the largest two outbreaks in Shanghai and Jilin (15, 16). Although all outbreaks have been completely controlled ultimately, markedly more ratios (26%) of Omicron outbreaks lasted longer (more than three maximal incubation times). Additionally, the proportion of fully vaccinated people in Omicron prevalent period was much higher than that of Delta. It reflects, from another perspective, the stronger transmissibility of Omicron VOCs.

Wide coverage of next-generation sequencing in public health units of China helps us trace the transmission sources and identify SARS-CoV-2 variants promptly during the outbreak (17, 18). Our data here reveal that approximately 57.3% of 124 Omicron outbreaks are associated with a single PANGO lineage subvariant, whereas the rest of the outbreaks are co-epidemic with 2 to 20 different subvariants. Large portions of Omicron outbreaks might indicate more than one transmission source in a special geographic region at a certain time or introduced with other new subvariant(s) during an outbreak. Co-circulations of more numbers of Omicron subvariants in an outbreak appeared to accompany prolonged duration and more affected cases, highlighting those wide circulations of viruses enhance the difficulty in outbreak containment.

Since the emergence of Omicron VOC in November 2021, nearly 400 evolutionary lineages and 40 recombinants have been described so far (13). Some subvariants within the BA.1, BA.2, BA.4, and BA.5 lineages show clear advantages in spreading globally (19). Coincidentally, more outbreaks are associated with BA.2 and BA.1 variants in the first half of 2022 and with BA.5 variants since July 2022. Given that the vaccine strains employed in mass vaccination campaign of China since 2021 predominantly consist of original strains, the heightened mutational profile observed in the Omicron variant, particularly within spike protein, makes them more capable of escaping immunity from natural infection and vaccination than previous variants. On the other hand, large-scale natural infection and widespread use of COVID-19 vaccines, including the booster doses of vaccines utilizing different technical approaches (20–22), produce large immunity pressure on the circulation of SARS-CoV-2, probably enhancing virus evolution. More numbers of mutations in virus genomes are frequently observed in relatively long-lasting outbreaks. Therefore, the rapid containment of Omicron outbreaks not only helps limit the spread but also contributes to delaying the virus mutation frequency.

## Conclusion

Based on a comprehensive analysis of the COVID-19 outbreaks, impacts, and evolution of SARS-CoV-2 variants in China, notable distinctions were observed between Delta and Omicron VOC-induced outbreaks. Furthermore, it was evident that the genome of SARS-CoV-2 continued to undergo evolutionary changes within and across outbreaks with different locations and time periods. These findings suggest that prompt containment measures for Omicron virus outbreaks can not only limit their geographical spread but also impede the frequency of viral mutations.

## Data Availability

The datasets presented in this study can be found in online repositories. The names of the repository/repositories and accession number(s) can be found in the article/supplementary material.

## References

[1] World Health Organization. WHO COVID-19 dashboard. Available at: https://data.who.int/dashboards/covid19/cases?n=c (Accessed September 23, 2024).

[2] Hu B Guo H Zhou P Shi ZL. Characteristics of SARS-CoV-2 and COVID-19. Nat Rev Microbiol. (2021) 19:141–54. doi: 10.1038/s41579-020-00459-7, PMID: 33024307 PMC7537588

[3] Li J Lai S Gao GF Shi W. The emergence, genomic diversity and global spread of SARS-CoV-2. Nature. (2021) 600:408–18. doi: 10.1038/s41586-021-04188-6 34880490

[4] World Health Organization. (2022). Available at: https://www.who.int/activities/tracking-SARS-CoV-2-variants (Accessed October 26, 2022).

[5] van Dorp L Houldcroft CJ Richard D Balloux F. COVID-19, the first pandemic in the post-genomic era. Curr Opin Virol. (2021) 50:40–8. doi: 10.1016/j.coviro.2021.07.002, PMID: 34352474 PMC8275481

[6] Liu Y Liu J Shi PY. SARS-CoV-2 variants and vaccination. Zoonoses. (2022) 2. doi: 10.15212/zoonoses-2022-0001, PMID: 35284912 PMC8909890

[7] Viana R Moyo S Amoako DG Tegally H Scheepers C Althaus CL. Rapid epidemic expansion of the SARS-CoV-2 omicron variant in southern Africa. Nature. (2022) 603:679–86. doi: 10.1038/s41586-022-04411-y, PMID: 35042229 PMC8942855

[8] World Health Organization. (2022). Available at: https://covid19.who.int (Accessed October 26, 2022).

[9] Feng Y Zhao X Chen Z Nie K Yin Z Xia Y. Genomic surveillance for SARS-CoV-2 variants of concern from imported COVID-19 cases - the mainland of China, 2021. China CDC Wkly. (2022) 4:680–4. doi: 10.46234/ccdcw2022.144, PMID: 36059791 PMC9433767

[10] Tan Z Chen Z Yu A Li X Feng Y Zhao X. The first two imported cases of SARS-CoV-2 omicron variant - Tianjin municipality, China, December 13, 2021. China CDC Wkly. (2022) 4:76–7. doi: 10.46234/ccdcw2021.266, PMID: 35186373 PMC8837443

[11] Global Epidemic Analysis and Risk Assessment Platform, China CDC. (2022). Available at: https://epiworld.chinacdc.cn/views/epidemic/global (Accessed August 15, 2022).

[12] Rambaut A Holmes EC O'Toole A Hill V McCrone JT Ruis C. A dynamic nomenclature proposal for SARS-CoV-2 lineages to assist genomic epidemiology. Nat Microbiol. (2020) 5:1403–7. doi: 10.1038/s41564-020-0770-5, PMID: 32669681 PMC7610519

[13] Pango Network. (2022). Available at: https://www.pango.network (Accessed October, 26 2022).

[14] Jacobs JL Haidar G Mellors JW. COVID-19: challenges of viral variants. Annu Rev Med. (2022) 74:31–53. doi: 10.1146/annurev-med-042921-020956 35850493

[15] Zhang X Zhang W Chen S. Shanghai's life-saving efforts against the current omicron wave of the COVID-19 pandemic. Lancet. (2022) 399:2011–2. doi: 10.1016/S0140-6736(22)00838-8, PMID: 35533708 PMC9075855

[16] Sun RJ Chen XH Wu YP Yu HJ. Factors associated with the clinical severity and disease burden of COVID-19 caused by omicron BA.2 in Shanghai and Hong Kong, China. Zoonoses. (2024) 4:9. doi: 10.15212/ZOONOSES-2023-0055

[17] John G Sahajpal NS Mondal AK Ananth S Williams C Chaubey A. Next-generation sequencing (NGS) in COVID-19: a tool for SARS-CoV-2 diagnosis, monitoring new strains and Phylodynamic modeling in molecular epidemiology. Curr Issues Mol Biol. (2021) 43:845–67. doi: 10.3390/cimb43020061, PMID: 34449545 PMC8929009

[18] Chen C Feng Y Chen Z Xia Y Zhao X Wang J. SARS-CoV-2 cold-chain transmission: characteristics, risks, and strategies. J Med Virol. (2022) 94:3540–7. doi: 10.1002/jmv.27750, PMID: 35355277 PMC9088485

[19] Chavda VP Balar P Vaghela D Solanki HK Vaishnav A Hala V. Omicron variant of SARS-CoV-2: an Indian perspective of vaccination and management. Vaccines (Basel). (2023) 11:160. doi: 10.3390/vaccines11010160, PMID: 36680006 PMC9860853

[20] Lin L Pei Y Li Z Luo D. Progress and challenges of mRNA vaccines. Interdiscip Med. (2023) 1:e20220008. doi: 10.1002/INMD.20220008 37096256

[21] Tregoning JS Flight KE Higham SL Wang Z Pierce BF. Progress of the COVID-19 vaccine effort: viruses, vaccines and variants versus efficacy, effectiveness and escape. Nat Rev Immunol. (2021) 21:626–36. doi: 10.1038/s41577-021-00592-1, PMID: 34373623 PMC8351583

[22] Hadj HI. Covid-19 vaccines and variants of concern: a review. Rev Med Virol. (2022) 32:e2313. doi: 10.1002/rmv.2313, PMID: 34755408 PMC8646685

