# Supplementary material for: COVID-19 outbreaks caused by different SARS-CoV-2 variants: a descriptive, comparative study from China
Source: Front Public Health. 2024 Dec 12;12:1416900. doi: 10.3389/fpubh.2024.1416900 (PMC11672794; doi:10.3389/fpubh.2024.1416900)
Supplement: Supplementary file 1 [file Table_1.DOCX]

Table 1. Numbers of increased nucleotide mutations of various subvariants of SARS-CoV-2 Delta and Omicron from the samples of the early and late cases in each outbreak

| WHO | PANGO  Lineage | Primary epidemic province | Total cases | No. of mutations in early cases* | Sampling date of early cases | No. of mutations in late cases | Sampling date of late cases* | No. of increased mutations. | Epidemic duration (d) |
| --- | --- | --- | --- | --- | --- | --- | --- | --- | --- |
| Omicron | BA.2.2.1 | Shanghai | 627109 | 74 | 2022/2/28 | 84 | 2022/6/25 | 10 | 171 |
|  | BA.2 | Liaoning | 4192 | 67 | 2022/3/7 | 71 | 2022/5/10 | 4 | 77 |
|  | BA.2.3 | Guangxi | 161 | 72 | 2022/7/8 | 75 | 2022/7/26 | 3 | 35 |
|  | BA.1.1 | Tianjin | 905 | 57 | 2022/1/8 | 59 | 2022/1/28 | 2 | 31 |
| Delta | AY.126 | Shaanxi | 2121 | 44 | 2021/10/19 | 47 | 2021/11/2 | 3 | 43 |
|  | B.1.617.2 | Fujian | 471 | 35 | 2021/9/9 | 38 | 2021/9/27 | 3 | 23 |
|  | AY.122 | Jiangsu | 1162 | 35 | 2021/7/22 | 37 | 2021/8/15 | 2 | 37 |
|  | AY.31 | Henan | 167 | 48 | 2021/8/9 | 49 | 2021/8/14 | 1 | 25 |

* Early and late case derived from the first and last batch sequenced cases from each outbreak.
