# Supplementary material for: COVID-19 outbreaks caused by different SARS-CoV-2 variants: a descriptive, comparative study from China
Source: Front Public Health. 2024 Dec 12;12:1416900. doi: 10.3389/fpubh.2024.1416900 (PMC11672794; doi:10.3389/fpubh.2024.1416900)
Supplement: Supplementary file 2 [file Table_2.DOCX]

Table 2. Viral genomic association analysis of early and late outbreaks based on nucleotide differences in various subvariants of SARS-CoV-2 Omicron.

| PANGO Lineage | Early outbreak | | | Late outbreak | | | Differential mutations |
| --- | --- | --- | --- | --- | --- | --- | --- |
|  | Primary epidemic province | Sampling date of presentative sequence | No. of mutations | Primary epidemic province | Sampling date of presentative sequence | No. of mutations | Increased |
| BA.2.2.1 | Beijing | 2022/4/24 | 74 | Hubei | 2022/8/8 | 78 | ORF1a: A2140G  ORF1a: C3874T  ORF1a: T4351C  ORF1a: C10432T |
| BA.2.2 | Shenzhen | 2022/2/28 | 69 | Shenzhen | 2022/6/18 | 76 | ORF1a: C3784T  ORF1a: G3980A  ORF1a: A4675G  ORF1a: T10696C  ORF1b: T17166A  ORF1b: C17397A  ORF3a: 25511T |
