# Supplementary figures and images for: COVID-19 outbreaks caused by different SARS-CoV-2 variants: a descriptive, comparative study from China
Source: Front Public Health. 2024 Dec 12;12:1416900. doi: 10.3389/fpubh.2024.1416900 (PMC11672794; doi:10.3389/fpubh.2024.1416900)

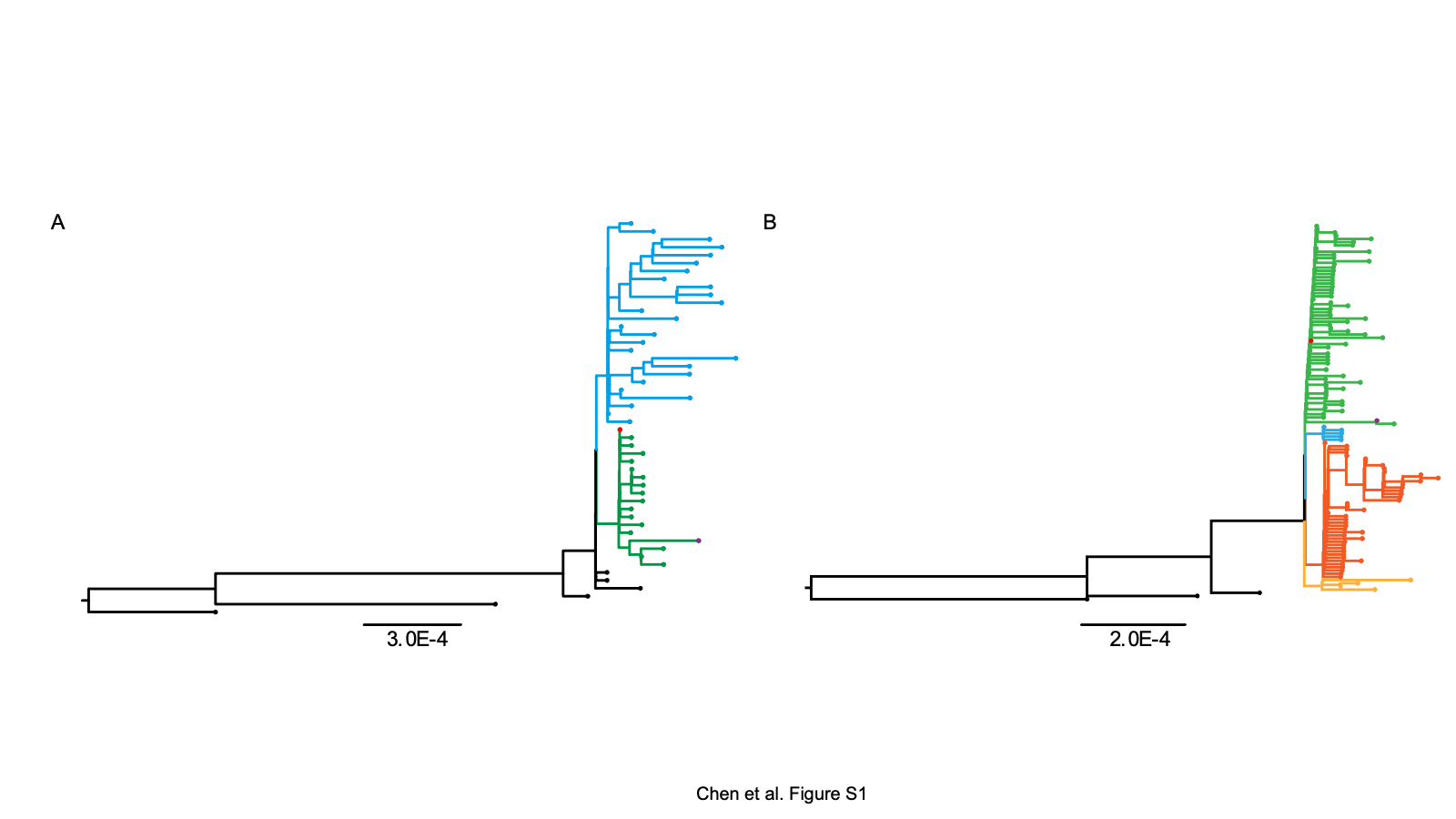

Supplement: Supplementary file 3 [file Image_1.TIFF]
